# Glutamate induces synthesis of thrombogenic peptides and extracellular vesicle release from human platelets

**DOI:** 10.1038/s41598-019-44734-x

**Published:** 2019-06-06

**Authors:** Deepa Gautam, Arundhati Tiwari, Rameshwar Nath Chaurasia, Debabrata Dash

**Affiliations:** 1 Department of Biochemistry, Institute of Medical Sciences, Banaras Hindu University, Varanasi, India; 2 Department of Neurology, Institute of Medical Sciences, Banaras Hindu University, Varanasi, India

**Keywords:** Cytoskeletal proteins, Ion channels in the nervous system

## Abstract

Platelets are highly sensitive blood cells, which play central role in hemostasis and thrombosis. Platelet dense granules carry considerable amount of neurotransmitter glutamate that is exocytosed upon cell activation. As platelets also express glutamate receptors on their surface, it is pertinent to ask whether exposure to glutamate would affect their signalling within a growing thrombus. In this study we demonstrate that, glutamate *per se* induced synthesis of thrombogenic peptides, plasminogen activator inhibitor-1 and hypoxia-inducible factor-2α, from pre-existing mRNAs in enucleate platelets, stimulated cytosolic calcium entry, upregulated RhoA-ROCK-myosin light chain/myosin light chain phosphatase axis, and elicited extensive shedding of extracellular vesicles from platelets. Glutamate, too, incited platelet spreading and adhesion on to immobilized matrix under arterial shear, raised mitochondrial transmembrane potential associated with generation of reactive oxygen species and induced activation of AMP-activated protein kinase in platelets. Taken together, glutamate switches human platelets to pro-activation phenotype mediated mostly through AMPA receptors and thus targeting glutamate receptors may be a promising anti-platelet strategy.

## Introduction

Platelets, sub-cellular fragments derived from megakaryocytes, circulate in blood as tiny discs of 2–4 µm diameters. During vascular injury platelets quickly adhere to exposed subendothelial proteins as well as to each other to form macroscopic aggregates that seal breach in endothelium^1^. However, pathological activation of platelets can lead to formation of occlusive thrombi accompanied with serious consequences like acute coronary syndrome, myocardial infarction, ischemic stroke and venous thromboembolism^2^. Platelets have considerable presence of glutamate in their dense granules^3,4^ coexistent with thrombogenic mediators like adenosine diphosphate (ADP), serotonin and Ca^2+ ^^5^, all of whom are exocytosed upon platelet activation. Secreted ADP, serotonin and thromboxane A2 (TXA2) bind to respective receptors on platelet surface and establish positive feedback loops to potentiate platelet stimulation^6^. Pharmacological inhibitors of ADP receptors and cyclooxygenase are among the first line of anti-platelet medications employed against cardiovascular disorders^7,8^. As platelets express glutamate receptors (see below) and serum glutamate rises locally between 400–800 µM during thrombus formation^9^, it is pertinent to ask whether glutamate can, too, behave in autocrine/paracrine manner similar to ADP and TXA2 to augment platelet stimulation and recruit fresh platelets in thrombus.

Glutamate is the main excitatory neurotransmitter in the mammalian cortex that plays critical role in functioning of brain^10^. Platelets are considered as peripheral model of neurons as both cell types share morphological and functional similarities attributable to their common ectodermic origin. Platelet membrane possesses two broadly classified glutamate receptors - metabotropic and ionotropic^11^. The former is the G protein-coupled receptor that recruits diacylglycerol and cAMP for signal amplification. Ionotropic glutamate receptors are ligand-gated ion channels permitting non-specific entry of cations, and are categorized into 3 subfamilies, N-methy-D-aspartate receptor (NMDAR), α-amino-3-hydroxy-5-methyl-4-isoxazole propionic acid receptor (AMPAR) and kainate receptor^12^. Although glutamate has been reported to potentiate agonist-induced platelet activation^9,12^, its effect on platelet signalling *per se* remains unclear.

We have recently reported pro-thrombotic attributes of amyloidogenic neurotoxic peptides like amyloid beta and prion protein^13,14^. In the present study we demonstrate that, glutamate switches human platelets to pro-activation phenotype as reflected from synthesis of thrombogenic peptides from pre-existing mRNAs, activation of RhoA-Rho kinase-myosin light chain (MLC) signalling axis, extensive shedding of extracellular vesicles (EVs), augmented spreading on immobilized matrix, and formation of large platelet microthrombi under arterial shear. Strikingly, AMPA receptor antagonist mitigates the thrombogenic effect of glutamate on platelets. Thus, targeting glutamate receptors combined with inhibition of cyclooxygenase and purinergic ADP receptors can be a potential anti-platelet therapeutic strategy.

## Results

### Glutamate induces rise in intracellular Ca^2+^ in platelets

**Figure 1:**
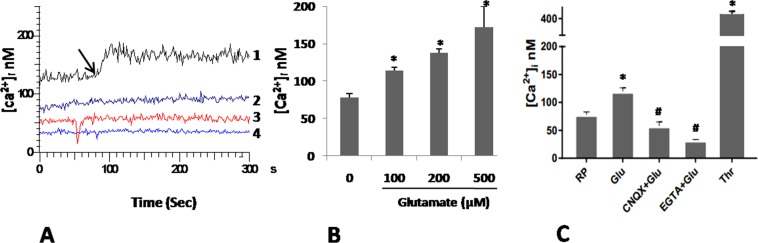
Glutamate raises free intracellular Ca^2+^ in human platelets. (**A**) Fura-2-loaded platelets were pre-incubated with either vehicle (tracing 1), or 100 μM CNQX (tracing 3), or 1 mM EGTA (tracing 4), followed by addition of 500 μM glutamate along with 10 µM glycine (indicated by arrow). Tracing 2 represents resting platelets without glutamate treatment. Ca^2+^ (1 mM) was included in all samples except experiments with EGTA. Corresponding values are graphically presented in (**C**). (**B**) Dose-dependent rise in intracellular calcium from glutamate-stimulated platelets. Results in (**B**,**C**) represent average of atleast 5 independent experiments (mean ± SEM). *P < 0.01 as compared to resting platelets (RP); ^#^P < 0.01 as compared to glutamate-stimulated platelets.

Cytosolic free Ca^2+^ is a critical regulator of platelet activity^15^. Incubation of platelets with increasing doses (100, 200 and 500 μM) of glutamate in presence of 1 mM Ca^2+^ led to significant rise in intracellular Ca^2+^ (from basal 78.21 ± 4.77 nM to 113.85 ± 4.91, 137.44 ± 5.31 and 172.27 ± 27.40 nM, respectively) (Fig. 1B), which dropped significantly following prior exposure to 100 µM L-Glutamic acid, 6-cyano-7-nitroquinoxaline-2,3-dione (CNQX), antagonist of AMPAR (Fig. 1A,C). In order to examine the source of raised intracellular calcium, we chelated external calcium with 1 mM ethylene glycol tetraacetic acid (EGTA) followed by addition of 500 μM glutamate. EGTA completely abolished glutamate-induced rise in intracellular Ca^2+^ (Fig. 1A,C), suggestive of calcium influx from external medium.

### Glutamate induces shedding of EVs from platelets

**Figure 2:**
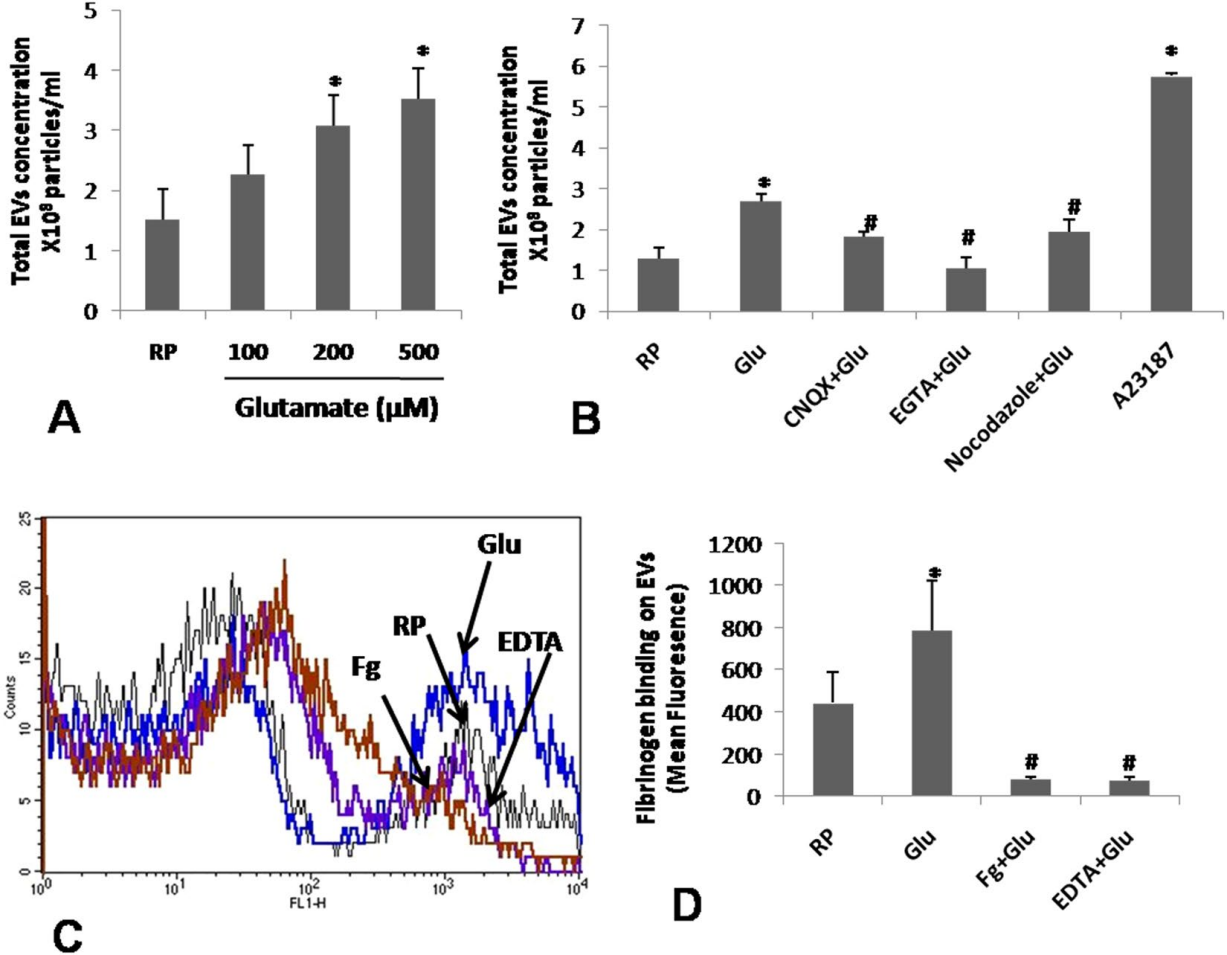
Glutamate induces generation of EVs from platelets. (**A**) Dose-dependent rise in EVs released from glutamate-stimulated platelets. (**B**) Release of EVs from platelets pre-treated with glutamate, CNQX, EGTA, nocodazole or A23187 as indicated. (**C**,**D**) Binding of fluorescent fibrinogen to platelets pre-treated with non-fluorescent fibrinogen, EDTA and vehicle as indicated. Bar diagrams represent atleast 5 independent experiments (mean ± SEM). *P < 0.03 as compared to resting platelets; ^#^P < 0.03 as compared to glutamate-stimulated platelets.

Platelets generate EVs when challenged with physiological agonsists like thrombin, calcium ionophore or under conditions of stress^16,17^. Exposure of platelets to 100 µM glutamate evoked release of 2.28 × 10^8^ ± 0.85 EVs/ml (in size range 100–250 nm, 90% of population being between 150–200 nm) from platelets, which was increased by 1.36- and 1.55-folds in presence of 200 and 500 µM glutamate, respectively (Fig. 2A). Remarkably, EVs released from glutamate-treated platelets bound Alexa fluor 488-labeled fibrinogen (Fig. 2C,D), suggestive of thrombogenic milieu prompted by glutamate. The binding was competitively inhibited when EVs were pre-incubated with non-fluorescent fibrinogen (10 µg/ml) or in presence of ethylene diamine tetraacetic acid (EDTA) (5 mM) (that dissociates the α_IIb_β_3_ integrins^18^) (by 96.82% ± 12.54 and 98% ± 17.20, respectively) (Fig. 2C,D).

As glutamate induced Ca^2+^ entry in platelets (Fig. 1), we subsequently studied its effect on shedding of EVs. When extracellular Ca^2+^ was chelated with EGTA, EV generation from glutamate-treated platelets was lowered by 63.88% ± 0.12 (Fig. 2B), suggestive of critical role of Ca^2+^ influx on release of EVs. Pre-treatment of platelets with CNQX (100 µM) and nocodazole (10 µM) (pharmacological inhibitor of microtubule polymerization), too, significantly attenuated glutamate-induced EV release by 37.15% ± 0.29 and 32.29% ± 0.26, respectively, implicating AMPAR ligation and microtubule reorganization in glutamate-mediated shedding of EVs (Fig. 2B).

### Glutamate instigates platelet spreading and aggregate formation under flow upon immobilized matrix

**Figure 3:**
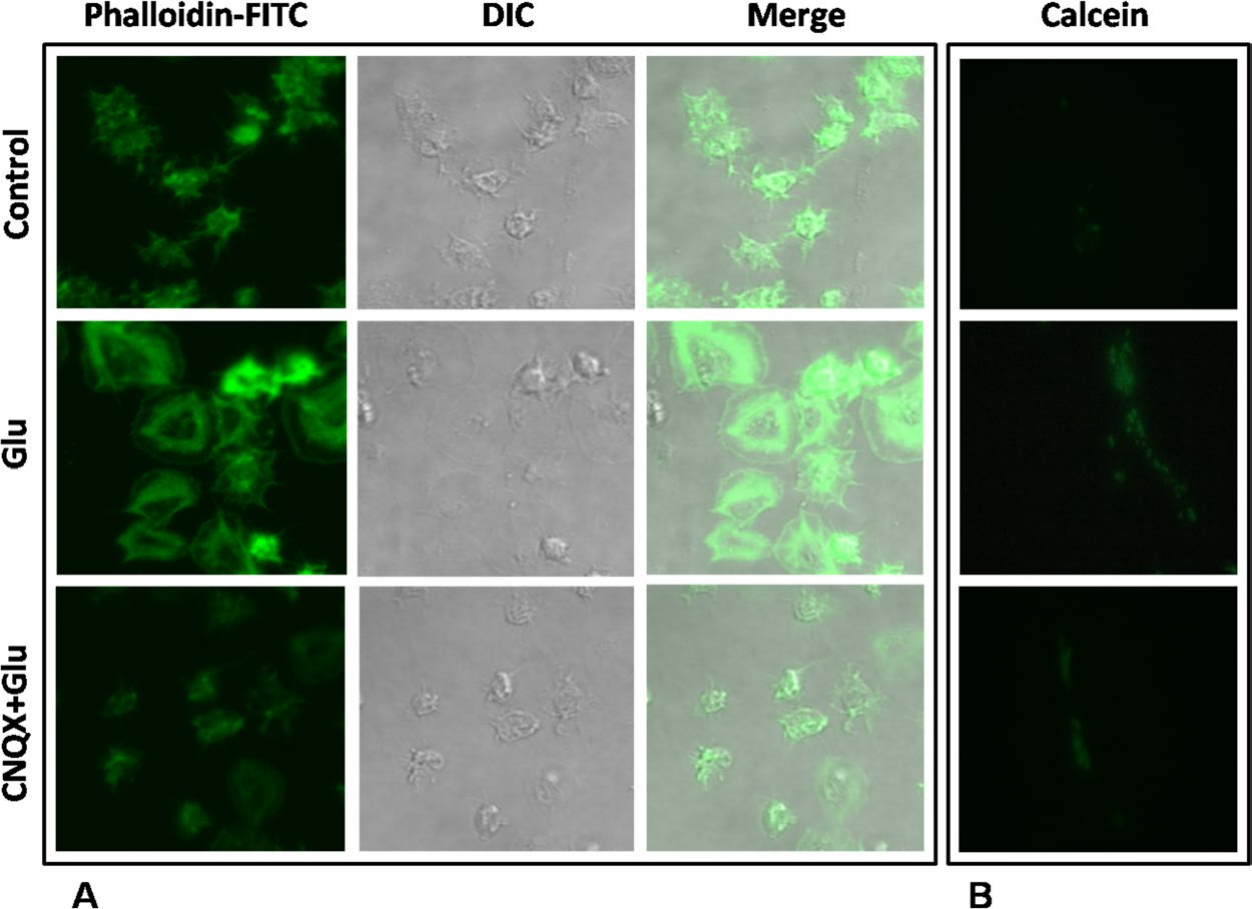
Glutamate evokes platelet spreading (under static condition) and aggregate/microthrombi formation (under arterial shear) on immobilized matrix. (**A**) Confocal images of phalloidin-FITC-labeled control and glutamate (500 µM)-treated platelets undergoing spreading upon immobilized fibrinogen under static condition in absence or presence of 100 µM CNQX as indicated. (**B**) Fluorescence microscopy images of calcein-stained control and glutamate (500 µM)-treated platelets adhered on to immobilized collagen under flow at shear rate 1500 s^−1^, in absence or presence of 100 µM CNQX as indicated. Figures are representative of images from 5 different fields of 3 independent experiments.

We next explored the effect of glutamate on adhesion signalling in human platelets as described for thrombin^18–20^. Platelets seeded on to immobilized fibrinogen underwent adhesion, followed by spreading with protrusion of filopodia/microspikes (Fig. 3A, upper panel). Although glutamate pre-treatment did not affect the number of cells adhered on to matrix, it strongly augmented the extent of platelet spreading with expression of prominent lamellipodia-like structures (Fig. 3A, middle panel), which was notably attenuated by glutamate receptor inhibitor CNQX (100 µM) (Fig. 3A, lower panel).

Next, a dynamic adhesion assay was implemented to analyse platelet immobilization upon collagen matrix under arterial shear (1500 s^−1^). Glutamate (500 µM) strongly stimulated cell adhesion under flow with formation of large microthrombi/aggregates in all fields studied (Fig. 3B, middle panel), which was significantly prevented by CNQX (100 µM) (Fig. 3B, lower panel).

### Glutamate induces RhoA activity in human platelets

**Figure 4:**
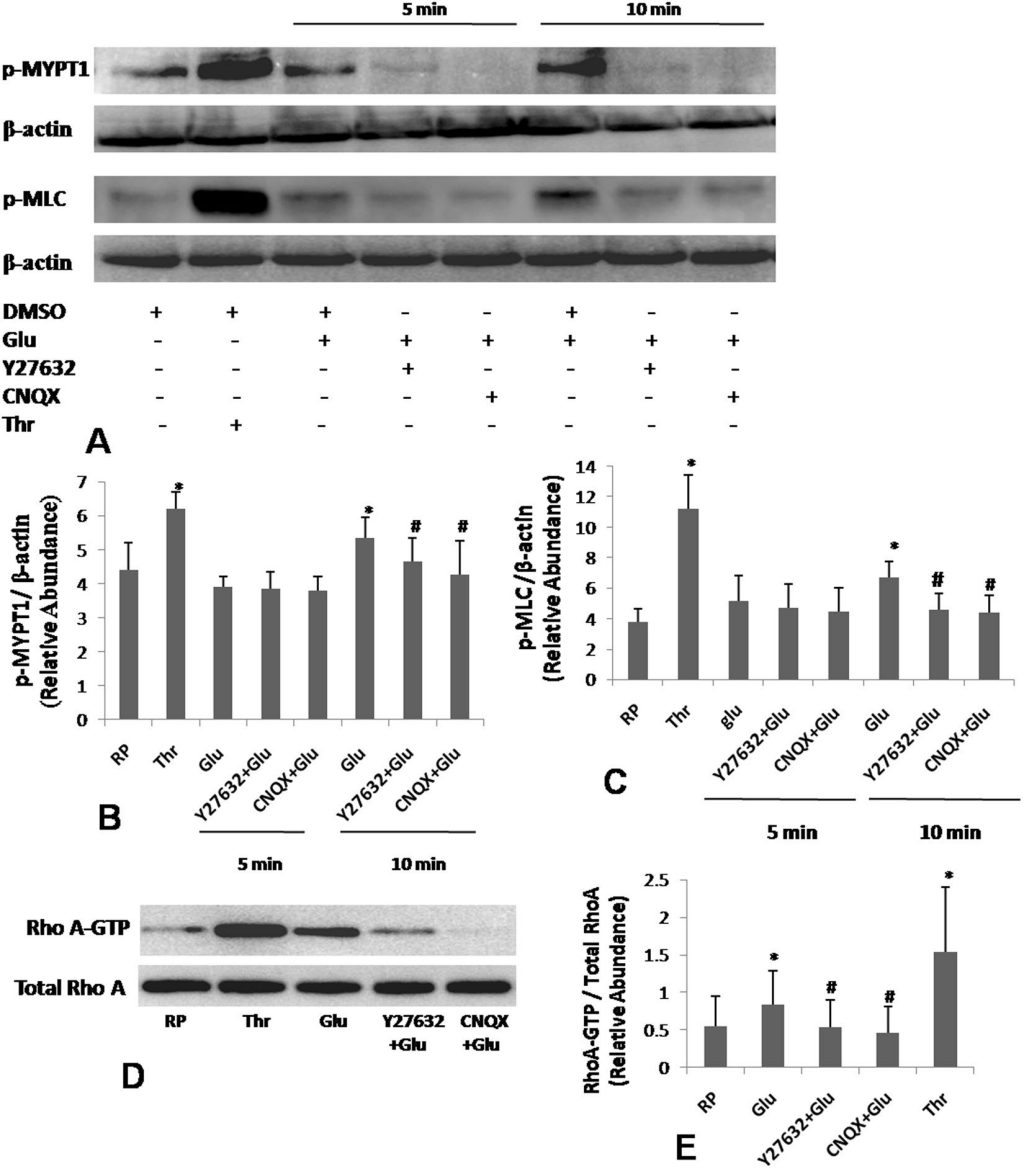
Glutamate activates RhoA-ROCK-MLC/MYPT1 pathway. (**A**) Platelets were stimulated with either thrombin (1 U/ml) for 10 min or 500 µM glutamate (for either 5 or 10 min, in presence or absence of 10 µM Y27632 and 100 µM CNQX) and immunoblotted against p-MYPT1 and p-MLC as indicated. (**B**,**C**) Densitometric analysis of immunoblots for p-MYPT1 and p-MLC, respectively, normalized with respect to β-actin. (**D**) Expression of RhoA-GTP in human platelets stimulated for 10 min with either thrombin or glutamate in presence or absence of Y27632 and CNQX as indicated. Fulllength blots for (**A**,**D**) are presented in Supplementary Figs S3 and S4, respectively. (**E**) Densitometric analysis of RhoA-GTP normalized against total RhoA expression. Bar diagrams represent atleast 5 independent experiments (mean ± SEM). *P < 0.03 as compared to resting platelets; ^#^P < 0.03 as compared to glutamate-stimulated platelets for p-MYPT1. *P < 0.05 as compared to resting platelets; ^#^P < 0.05 as compared to glutamate-stimulated platelets for p-MLC and RhoA-GTP.

We next studied the effect of glutamate on activation of RhoA-ROCK-MLC/MLC phosphatase (MYPT1) pathway, which regulates remodelling of platelet cytoskeleton^13,19^. Exposure of platelets to 500 µM glutamate for 5 min did not elicit any significant change in phosphorylation of MLC and MYPT1. However, following 10 min exposure, of glutmate induced phosphorylation of MLC and MYPT1 by 87.79% ± 2.34 and 21.31% ± 3.38, respectively (Fig. 4A–C), which was reversed by 10 µM Y27632, a pharmacological inhibitor of ROCK (by 31.13% ± 2.04 and 21.5% ± 3.29, respectively), as well as by AMPA receptor inhibitor CNQX (by 22.72% ± 2.28 and 20.56% ± 3.12, respectively) (Fig. 4A–C). As RhoA regulates phosphoryaltion of MLC/MYPT1 through effector kinase ROCK, we subsequently evaluated RhoA activity in glutamate-treated platelets by pulldown assay. Glutamate elicited significant rise in expression of RhoA-GTP (by 53.21% ± 0.79) in platelets, which was abrogated upon pre-treatment with either Y27632 or CNQX (by 36.16% ± 0.63 and 45.38% ± 0.61, respectively) (Fig. 4D,E). These observations were consistent with activation of RhoA-ROCK-MLC/MYPT1 axis downstream of AMPA receptor in platelets challenged with glutamate.

### Glutamate induces synthesis of hypoxia-inducible factor-2α (HIF-2α) and plasminogen activator inhibitor-1 (PAI-1) in human platelets

**Figure 5:**
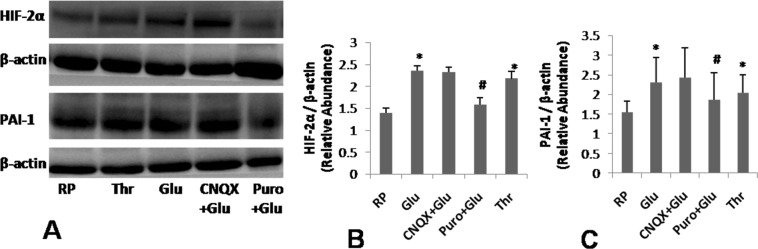
Glutamate induces synthesis of thrombogenic peptides in platelets. (**A**) Immunoblots representing expression of HIF-2α and PAI-1 in washed human platelets stimulated with either thrombin (1 U/ml) or glutamate (500 µM) in presence or absence of 100 µM CNQX and 10 mM puromycin for 30 min. Uncropped blots are presented in Supplementary Fig. S5. (**B**,**C**) Densitometric analysis of immunoblots for HIF-2α and PAI-1, respectively, normalized with respect to β-actin. Bars represent atleast 5 independent experiments (mean ± SEM). *P < 0.05 as compared to resting platelets; ^#^P < 0.05 as compared to glutamate-stimulated platelets.

We have earlier demonstrated that, platelets synthesize HIF-2α through an oxygen-independent non-canonical path when challenged with physiological agonists like thrombin^21^. We asked now whether glutamate, too, can stimulate synthesis of HIF-2α from pre-existing mRNA in enucleate platelets. Glutamate (500 µM) increased expression of HIF-2α in platelets by 70.28% ± 0.11 (Fig. 5A,B). Plasminogen activator inhibitor-1 (PAI-1), which stabilizes fibrin-rich thrombus, is a target gene of HIF-2α in renal carcinoma cells^20^. It has been demonstrated that, platelets synthesize PAI-1 when stimulated with agonists including thrombin^22^. Remarkably, we found that glutamate was able to induce translation of PAI-1 by 49.03% ± 0.63 in platelets (Fig. 5A,C) in parallel with expression of HIF-2α. Both synthesis of HIF-2α and PAI-1 were inhibited by 10 mM puromycin, pharmacological inhibitor of protein translation, by 32.76% ± 0.16 and 19.48% ± 0.70, respectively (Fig. 5A–C). CNQX did not attenuate synthesis of HIF-2α and PAI-1 in platelets (Fig. 5A–C), which excluded participation of AMPA receptors in this glutamate-specific response.

### Glutamate affects mitochondrial function

**Figure 6:**
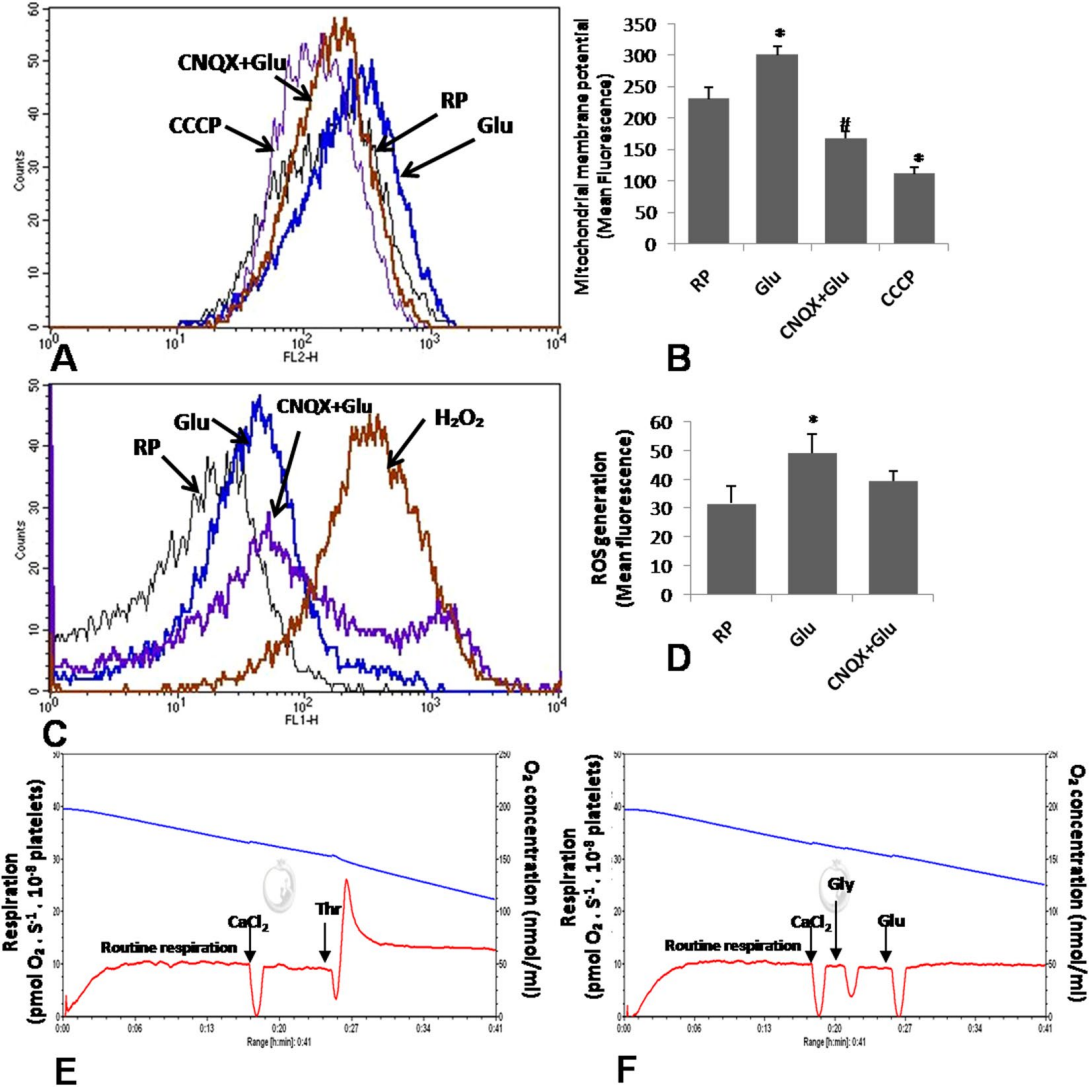
Glutamate affects platelet mitochondrial function. (**A**,**B**) Measurement of mitochondrial transmembrane potential by MitoTracker Red in glutamate (500 µM)-treated platelets in presence or absence 100 µM CNQX. CCCP (100 µM) was employed as control to dissipate ΔΨ. (**C**,**D**) Glutamate-induced rise in intracellular ROS in absence or presence of CNQX as indicated. H_2_O_2_ (10 µM) was used as control to raise ROS in platelets. (**E**,**F**), polarograms exhibiting oxygen flux in human platelets stimulated either with thrombin (1 U/ml) or glutamate (500 µM). Blue line represents oxygen concentration within the chamber while red line traces rate of oxygen consumption by the cells. Bars represent atleast 5 independent experiments (mean ± SEM). *P < 0.05 as compared to resting platelets; ^#^P < 0.05 as compared to glutamate-stimulated platelets.

Glutamate has been reported to modulate mitochondrial transmembrane potential (ΔΨ) and basal cellular respiration in murine hippocampal HT22 cells^23^. In the present study glutamate significantly enhanced ΔΨ in MitoTracker Red-treated platelets by 30.43% ± 3.89 (Fig. 6A,B). Glutamate also enhanced ROS yield in platelets by 55.66% ± 6.57 (Fig. 6C,D), which can be attributed to mitochondrial membrane hyperpolarization. CNQX normalized glutamate-induced rise in ΔΨ by 44.33% ± 12.1 (Fig. 6A,B). As expected, protonophore 100 µM carbonyl cyanide m-chlorophenyl hydrazine (CCCP) used as control dissipated mitochondrial transmembrane potential. To evaluate the effect of glutamate on mitochondrial respiratory function, we measured mitochondria oxygen comsumption rate using Clark amperometric electrode at high resolution (sampling at 2 s intervals). Glutamate, however, had no stimulatory effect on platelet oxygen consumption (Fig. 6F), which was consistent with hyperpolarized state of mitochondrial membrane in a coupled system.

**Figure 7:**
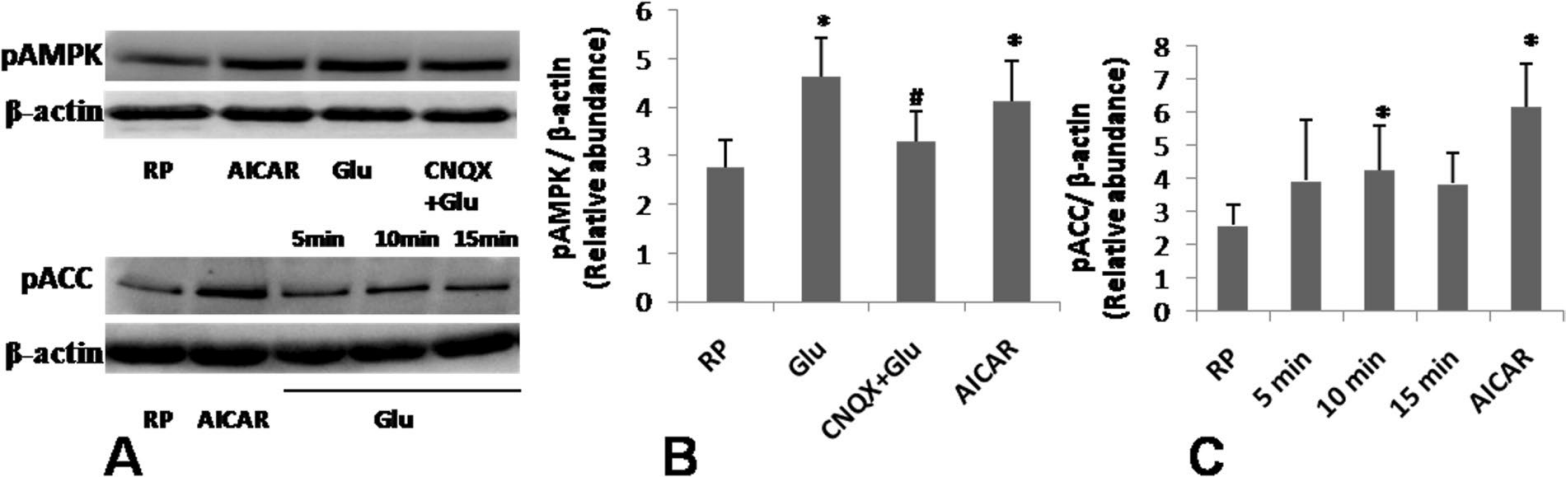
Glutamate stimulates AMPK and ACC in platelets. (**A**) Immunoblots representing expression of pAMPK (upper panel) or pACC (lower panel), in human platelets stimulated with either 1 mM AICAR, or 500 µM glutamate as indicated. (**B**,**C**) Corresponding densitometric analysis of immunoblots for pAMPK and pACC, normalized against β-actin expression. Fulllength blots for pAMPK and pACC are presented in Supplementary Fig. S6. Bars represent atleast 5 independent experiments (mean ± SEM). *P < 0.03 as compared to resting platelets; ^#^P < 0.03 as compared to glutamate-stimulated platelets for pAMPK and *P < 0.05 as compared to resting platelets; ^#^P < 0.05 as compared to glutamate-stimulated platelets for pACC.

As glutamate induced RhoA activation and MLC phosphorylation in platelets (Fig. 4) in absence of upsurge in mitochondrial respiration (Fig. 6F), this could lead to drop in energy charge and activation of AMP-activated protein kinase (AMPK), the energy sensor in the cell, in glutamate-treated platelets. Keeping with this, glutamate was found to significantly increase phosphorylation of AMPK (by 67.50% ± 1.03) (Fig. 7A, upper panel), suggestive of enhanced activity of the enzyme in platelets, which was inhibited by CNQX (by 28.66% ± 1.43). In consistence, glutamate also significantly enhanced phosphorylation (by 65.11% ± 1.34) of acetyl CoA carboxylase (ACC), an AMPK substrate, following 10 min incubation with platelets (Fig. 7A, lower panel). As a positive control 1 mM 5-Aminoimidazole-4-carboxamide ribonucleotide (AICAR), an AMP analog, was employed to stimulate AMPK. These results were supportive of state of relative ATP depletion in glutamate-treated platelets consistent with hyperpolarization of mitochondrial membrane.

## Discussion

In the present study we have demonstrated that, neurotransmitter glutamate can modulate platelet signalling *per se* in the absence of physiological agonists. Glutamate elicited extensive shedding of EVs from platelets, induced platelet adhesion on to immobilized collagen under arterial shear and cell spreading, provoked Ca^2+^ entry from external medium and stimulated synthesis of thrombogenic peptides, PAI-1 (that stabilizes fibrin-rich thrombus) and HIF-2α, all being hallmarks of pro-activation phenotype. Although PAI-1 is a target gene of HIF-2α in renal cell carcinoma^24^, platelets lack genomic DNA and have limited capacity to synthesize peptides. Glutamate has been shown to stimulate HIF translation in TNBC cell lines^25^. We have recently demonstrated that, platelets express HIF-2α when challenged with thrombin^21^. Strikingly, present observation adds glutamate to the existing list of agonists that induce protein synthesis in enucleate platelets.

As small GTPase RhoA is a critical regulator cytoskeletal reorganization in activated platelets, we probed RhoA-mediated signalling in cells challenged with glutamate. Pre-treatment with glutamate-stimulated phosphorylation of MLC and MYPT1, which were reversed by antagonists of ROCK (Rho effector kinase) as well as AMPAR. Consistent with this, glutamate potentiated GTP-loading of RhoA in platelets that validated activation of RhoA-ROCK-MLC/MYPT1 axis downstream of glutamate-AMPAR interaction. As glutamate was not found to stimulate mitochondrial oxygen flux, we predicted relative ATP depletion in glutamate-treated platelets. In agreement with this, activity of AMPK was found to be upregulated in platelets by glutamate.

Thrombin is known to activate RhoA-MLC axis^26^, release EVs^27^, induce synthesis of PAI-1^28^/HIF-2α^21^ and activate AMPK^29^ in platelets. Glutamate has earlier been shown to potentiate agonist-induced platelet activity^9,12^. Here we demonstrate that, glutamate *per se* at concentrations up to 500 µM brought about synthesis of thrombogenic peptides and extensive modulation in platelet signalling, mediated mostly through AMPA receptors, thus switching cells to pro-thrombotic phenotype. Glutamate, however, was neither able to induce platelet aggregation nor binding of PAC-1 (that specifically identifies the active conformation of platelet membrane integrins α_IIb_β_3_) at above concentrations (Supplementary Figs S1 and S2, respectively). Thus, we infer that glutamate did not incite inside-out signalling mediated through platelet integrins that would have led to fibrinogen binding and aggregation.

Plasma glutamate is elevated under neurological pathologies like blood-brain barrier breakdown^30^, autism^31^ and ischemic stroke^32^. Glutamate is also released from platelets in excess of 400 µM (in the concentration range 400–800 µM) into plasma during thrombus formation^3,9,12^. As glutamate could establish positive feedback loops to potentiate platelet stimulation in autocrine/paracrine manner similar to ADP and TXA2, targeting glutamate signalling in combination with established anti-platelet regimens may be a plausible therapeutic option.

## Material and Methods

### Materials

L-Glutamic acid, CNQX, AICAR, calcium ionophore A23187, CCCP, 2′, 7′-dichlorodihydrofluorescein diacetate (H_2_DCFDA), phalloidin-FITC, EGTA, EDTA, sodium orthovanadate, acetylsalicylic acid, thrombin, fibrinogen, dimethylsulfoxide (DMSO), anti-β-actin antibody (Ab) and Triton X-100, were purchased from Sigma. Fura 2/AM and anti-PAI-1 Ab were acquired from Calbiochem. Calcein-AM was from Life Technologies. Collagen was acquired from Chrono-log. Glycine and reagents for electrophoresis were from Merck. Polyvinylidene fluoride (PVDF) membranes and enhanced chemiluminescence detection kit were from Millipore. Antibodies against phospho(Thr-18/Ser-10)-myosin light chain (pMLC) and phospho(Thr-853)-MLC phosphatase (pMYPT1), phospho(Thr-172)-AMP-activated protein kinase (pAMPK), phospho(ser-79)-acetyl CoA carboxylase (pACC) were procured from Cell Signalling Technology. HIF-2α Ab was obtained from Novus Biochemicals. FITC-labeled PAC-1 and flow cytometry sheath fluid was from BD Biosciences. Sources of other reagents and antibodies used were as follow: Alexa fluor 488-fibrinogen and MitoTracker-Red (Invitrogen), horseradish peroxidase (HRP)-labeled anti-rabbit IgG (Bangalore Genei), and RhoA activation assay Biochem kit (Cytoskeleton). All other reagents were of analytical grade. Type I deionized water (18.2 MΩ⋅cm, Millipore) has been used throughout the experiments. Platelets were isolated from venous blood collected from healthy volunteers under informed consent, strictly as per the recommendations and as approved by the Institutional Ethical Committee of Banaras Hindu University. The study methodologies conformed to the standards set by the Declaration of Helsinki.

### Methods

#### Platelet Preparation

Platelets were isolated from fresh human blood by differential centrifugation, as already described^13^. Briefly, blood was collected from antecubital veins of healthy donors and centrifuged at 200 × g for 10 min. Platelet-rich plasma (PRP) was collected carefully to avoid the contamination of red and white blood cells and was incubated with 1 mM acetylsalicylic acid at 37 °C for 15 min. EDTA (5 mM) was added to PRP and was centrifuged at 600 × g for 10 min. Platelets pellet was washed in buffer A (20 mM HEPES, 138 mM NaCl, 2.9 mM KCl, 1 mM MgCl_2_, 0.36 mM NaH_2_PO_4_ and 1 mM EGTA, supplemented with 5 mM Glucose, pH 6.2) and centrifuged at 600 × g for 10 min. Platelet pellet was finally resuspended in buffer B (pH 7.4), which was the same as buffer A but without EGTA. Platelet count was adjusted to 2–4 × 10^8^/ml with Beckman Coulter Multisizer 4. Precautions were taken for asepsis and to maintain the cells in resting condition.

#### Measurement of cytosolic free Ca^2+^ in platelets

PRP was incubated with fura-2/AM at 37 °C for 45 min. Fura-2 loaded platelets were washed and suspended in buffer B. Fluorescence was taken in non-stirring condition in 400 µl aliquots of platelets at 37 °C using Hitachi fluorescence spectrophotometer (model F-2500). Fura-2 was excited at 340 and 380 nm and the emission wavelength was kept at 510 nm. Intracellular free Ca^2+^ concentration, [Ca^2+^]_i_ changes were monitored from the fluorescence ratio (340/380) using intracellular cation measurement program in FL solutions software, as described earlier^33^. F_max_ was determined by lysing platelets with 250 µM digitonin in presence of saturating CaCl_2_. F_min_ was determined by adding 2 mM EGTA. Intracellular free calcium was calibrated according to derivation of Grynkiewicz *et al*.^34^.

#### Study of extracellular vesicle release from platelets

Washed human platelets were treated with glutamate (500 µM) for 15 min at 37 °C. Cells were sedimented by centrifugation. Supernatant containing EVs was separated and were fixed with equal volume of 4% paraformaldehyde (PFA). Fixed supernatant was characterized by nanoparticle tracking analysis (NTA) where a beam from solid-state laser source (635 nm) was allowed to pass through the sample. 20 X microscope was used to observe the light scattered by rapidly moving particles in suspension in Brownian motion at room temperature (RT). Stokes Einstein equation was used to unveil the hydrodynamic diameter of particles, within range of 10 nm to 1 µm and concentration between 10^7^–10^9^/ml. The average distance moved by each EVs in x and y directions were captured with CCD camera (30 frames per sec) attached to the microscope. Both capture and analysis were performed using NTA 2.1 analytical software, which provides an estimate of the particle size versus concentration in sample.

In order to study fibrinogen-binding to integrin α_IIb_β_3_ expressed on EVs surface, suspension of EVs (100 µl) was incubated with Alexafluor488-conjugated fibrinogen (10 µg/ml) for 30 min in dark at room temperature. EVs were next fixed with equal volume of 4% PFA, washed and resuspended in sheath fluid. Samples were analyzed on flow cytometer as described earlier^13^.

#### Static adhesion and spreading of platelets on immobilized fibrinogen

Glass slides were coated with 100 µl fibrinogen (100 µg/ml) for 2 h, followed by addition of 100 µl bovine serum albumin (10 mg/ml) for 1 h. Washed human platelets (10^7^ cells/ml) were pre-treated with glutamate (500 µM) in presence or absence of CNQX (100 µM) and overlaid on fibrinogen-coated slides for 15 min. Cells were fixed with 100 µl PFA (4%) for 30 min, followed by three washing with PBS. Cells were permeabilized with 0.01% Triton X-100 for 30 s, followed by washing thrice with PBS. Permeabilized platelets were incubated with phalloidin-FITC (1 µM) for 15 min. Adhered cells were examined under Zeiss LSM 700 laser scanning confocal microscope with 63X objective and 1 AU pinhole size. Images were acquired and analyzed using ZEN imaging software as described earlier^13^.

#### Dynamic adhesion of platelets on immobilized collagen under flow

Washed human platelets were incubated with FITC-labeled calcein-AM (2 µg/ml) for 30 min at 37 °C. Cells were sedimented at 600 × g for 10 min followed by resuspension in platelet- poor plasma. Glass cover slip coated with Type I collagen was congregated in a parallel plate flow chamber (GlycoTech) and was mounted on the stage of an inverted epifluorescence video microscope (Nikon model Eclipse Ti-E) equipped with a monochrome CCD cooled camera. Syringe pump (Pump 22 infusion/withdrawal with standard syringe holder, Harvard Apparatus) was used to perfuse control and glutamate (500 µM)-treated platelets, in presence or absence of 100 µM CNQX, through the chamber at constant flow to yield wall shear rate 1500 s^−1^ (15 dyn/cm^2^). Images of fluorescent platelets from at least 5 different fields from each group were captured with DS-QiMC digital camera using NIS-Elements AR imaging software (Nikon).

#### Immunoblotting

Platelet proteins were separated on 10% sodium dodecyl sulfate polyacrylamide gel electrophoresis (SDS-PAGE gels) and electrophoretically transferred to PVDF membrane by using a semidry blotting system (BioRad). Membranes were blocked with 5% non-fat dry milk in Tris-buffered saline (10 mM Tris-HCl and 150 mM NaCl, pH 8.0) containing 0.05% Tween-20 (TBST) for 1 h at room temperature. Blots were incubated overnight with respective primary antibodies (p-MYPT1, 1:1000; p-MLC, 1:500; anti-RhoA, 1:500; anti-HIF-2α, 1:500; anti-PAI-1, 1:100; anti-pAMPK, 1:1000; anti pACC, 1:1000; anti-β-actin, 1:5000), followed by 3 washings with TBST for 5 min each. Membranes were then placed in HRP-labeled anti-rabbit IgG diluted in blocking buffer or TBST for 1 h. Blots were similarly washed, and antibody binding was detected using enhanced chemiluminescence. Images were acquired on a multispectral imaging system (BioSpectrum 800 Imaging system, UVP) and quantified using VisionWorks LS software (UVP)^13^.

#### RhoA-GTP pulldown assay

The assay was performed using a kit (Cytoskeleton) and following manufacturer′s instructions as described previously^13^. Washed human platelets, pre-treated with either glutamate (500 µM) or thrombin (1 U/ml) were lysed. Supernatant was incubated with 15 µl Rhotekin-Rho binding domain (Rhotekin-RBD) beads at 4 °C for 1 h. Samples were subjected to SDS-PAGE, western blotted and probed with mouse anti-human RhoA antibody followed by goat anti-mouse anti-IgG (1:20,000), as mentioned earlier^13^.

#### ΔΨ_M_ measurement

For elucidating ΔΨ_M_, washed human platelets were treated with glutamate in presence or absence of CNQX, followed by incubation with MitoTracker Red (500 nM) for 45 min. FL2 fluorescence was measured using flow cytometer^35^.

#### Measurement of intracellular ROS

Intracellular ROS was determined using a redox-sensitive cell-permeable dye, H_2_DCF-DA^36^. Washed human platelets were incubated at 37 °C for 5 min without stirring in the presence of glutamate (500 µM). H_2_DCF-DA (1 µM) was added to each sample and incubated for 30 min in the dark at RT. Platelets were fixed with 2% PFA. Cells were washed twice with PBS and resuspended in sheath fluid, followed by flow cytometry as described above. Hydrogen peroxide (1%) was added to platelet suspension as positive control.

#### Quantification of PAC-1 binding

Platelets on stimulation changes surface integrins α_IIb_β_3_ to an open conformation that binds to fibrinogen with high affinity and leads to platelets aggregation^37^. PAC-1 antibody recognizes open conformation of α_IIb_β_3._ Washed human platelets were stimulated with 500 µM glutamate at 37 °C for 10 min in non-stirring condition, followed by incubation with PAC-1 antibody (1.25 µg/ml) for 30 min in dark at room temperature. Platelets were fixed with equal volume of 4% paraformaldehyde for 20 min, washed twice with PBS and was resuspended in sheath fluid. Samples were analyzed with flow cytometer as described earlier^13^.

#### Platelet aggregation

Washed human platelets suspended in buffer B were stirred at 1200 rpm in optical lumi-aggregometer (Chrono-log model 700-2) at 37 °C for 1 min, followed by addition of thrombin (1 U/ml) or glutamate (200 and 500 µM) and transmittance was recorded. Aggregation was measured as percentage change in light transmission where 100% transmittance refers to transmittance through blank buffer solution^13^.

#### High-resolution respirometry for mitochondrial respiration

Mitochondrial respiration was measured using a high-resolution respirometer (Oxygraph-2k; Oroboros Instruments) at 37 °C under stirring conditions (750 rpm) as previously described^13^. Washed human platelets (in buffer B containing 5.5 mM glucose) with or without pre-treatment were transferred into oxygraph chamber. Respiration was first allowed to stabilize at the routine state, i.e., in the physiological coupling state controlled by cellular energy demands for oxidative phosphorylation. Then, platelets were treated with either glutamate (500 µM) or thrombin (1 U/ml), and changes in oxygen flux were recorded in real time at high resolution (sampling at 2 s intervals). Calibration at air saturation was performed each day before starting experiments by letting Millipore water/buffer B stir with air in the oxygraph chamber until equilibration and a stable signal was obtained. All experiments were performed at an oxygen concentration in the range of 100–205 µM O_2_. Data were recorded and analyzed using DatLab 5.1 software (Oroboros Instruments)^13^.

#### Statistical methods

Standard statistical methods were utilized to present the data. Two-tailed Student’s *t* test was employed for evaluation of significance. Tests were considered significant at *P* < 0.05. All data are presented as mean ± SEM of ≥3 individual experiments.

## Supplementary information

Dataset 1
