# Supplementary material for: Glutamate induces synthesis of thrombogenic peptides and extracellular vesicle release from human platelets
Source: Sci Rep. 2019 Jun 6;9:8346. doi: 10.1038/s41598-019-44734-x (PMC6554302; doi:10.1038/s41598-019-44734-x)
Supplement: Supplementary file 1 — Dataset 1 [file 41598_2019_44734_MOESM1_ESM.docx]

**Glutamate induces synthesis of thrombogenic peptides and extracellular vesicle release from human platelets**

Deepa Gautam^1, 2^, Arundhati Tiwari^1^, Rameshwar Nath Chaurasia^2^, Debabrata Dash^1^*

^1^Department of Biochemistry, Institute of Medical Sciences, Banaras Hindu University, Varanasi, India

^2^Department of Neurology, Institute of Medical Sciences, Banaras Hindu University, Varanasi, India

***Correspondence**: Debabrata Dash, Department of Biochemistry, Institute of Medical Sciences, Banaras Hindu University, Varanasi, India, Tel: +91 9336910665, Fax: +91 542 2367568, E-mail: [ddash.biochem@gmail.com](mailto:ddash.biochem@gmail.com)


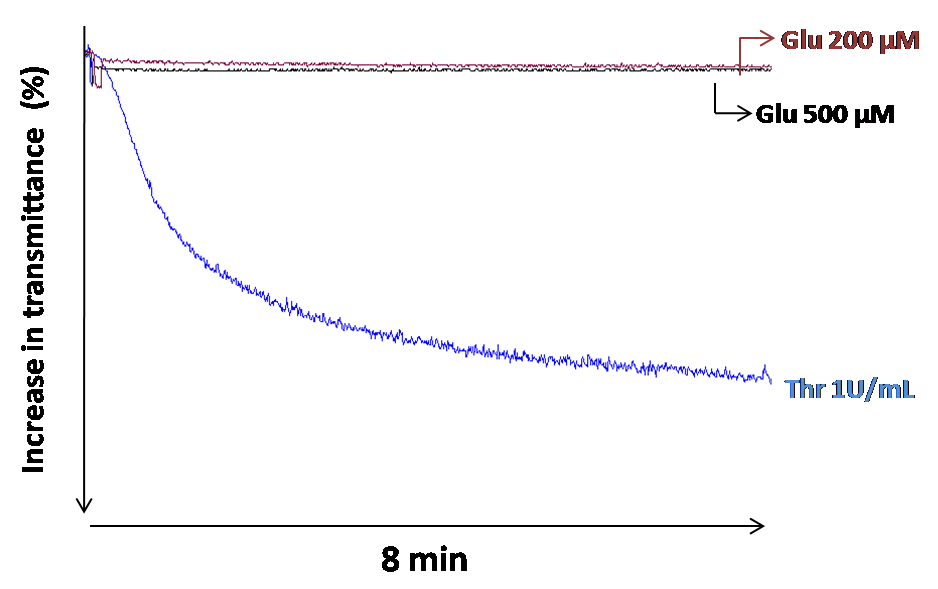


Supplementary Figure S1. Platelet aggregation induced by thrombin (1 U/ml) and glutamate (200 and 500 µM).


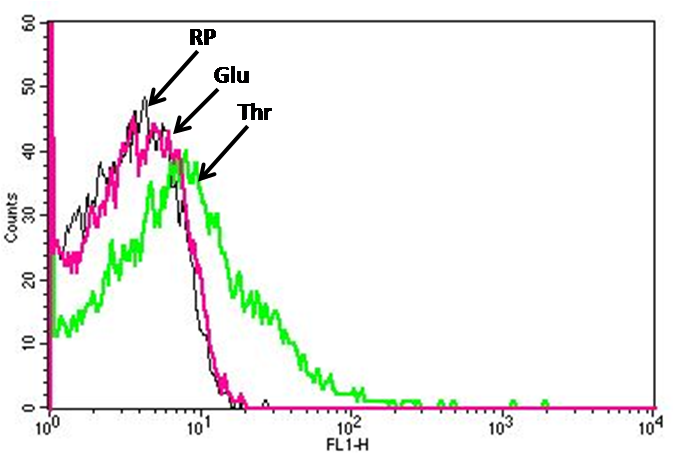


Supplementary Figure S2. Flow cytometry representing binding of PAC-1-FITC to platelets.


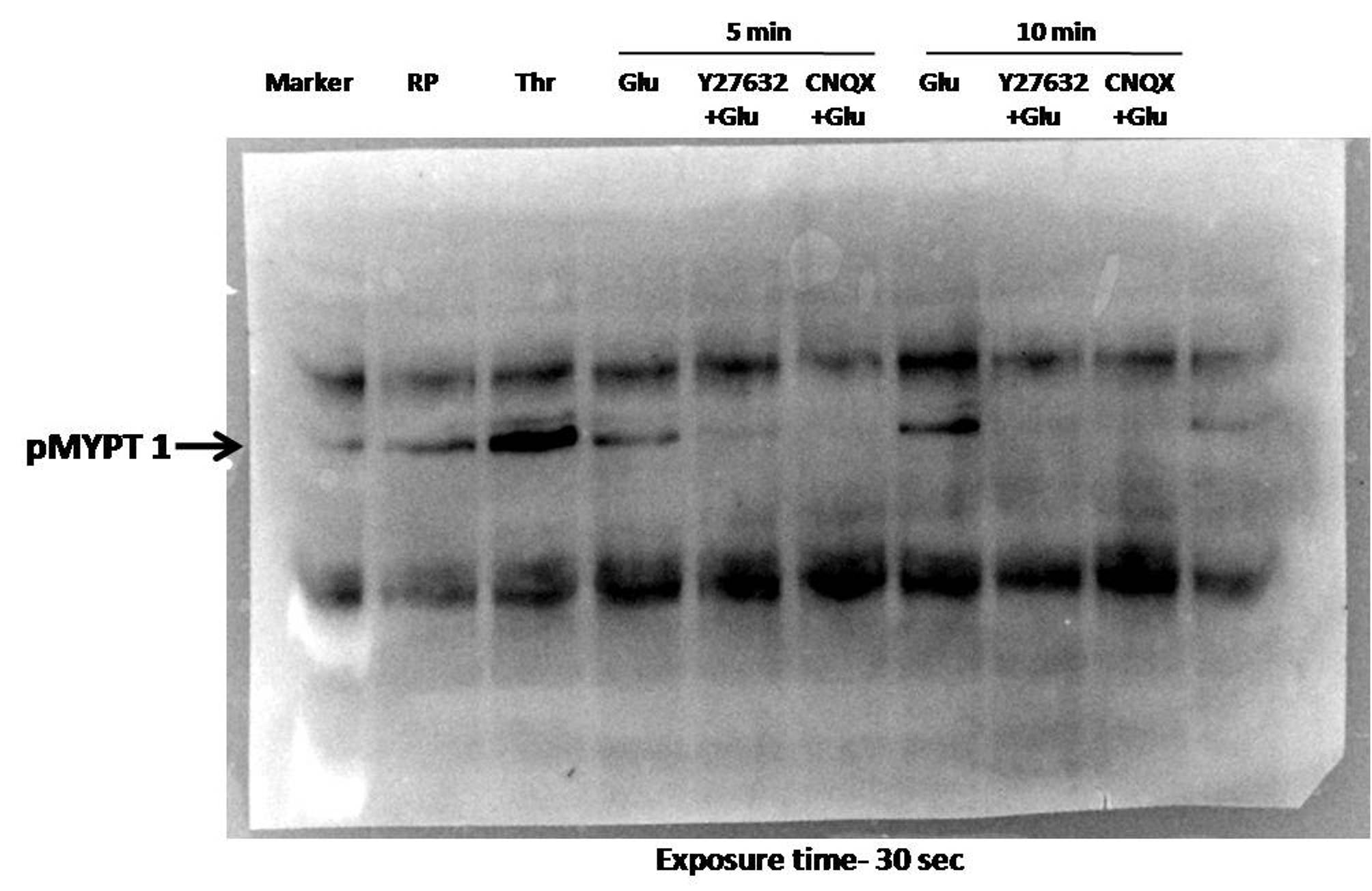

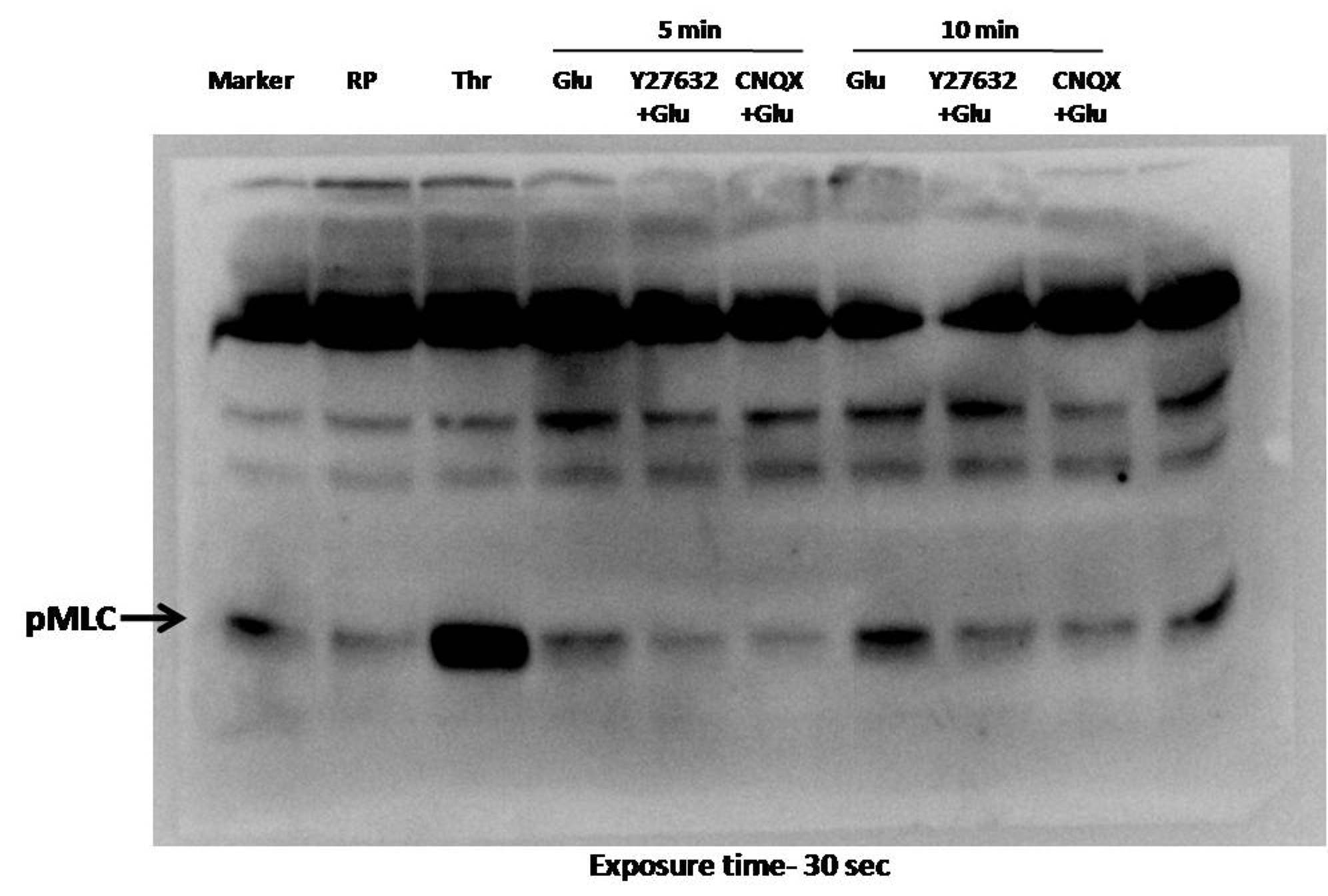


Supplementary Figure S3. Uncropped blots of Figure 4A against pMYPT1 (upper panel) and pMLC (lower panel).


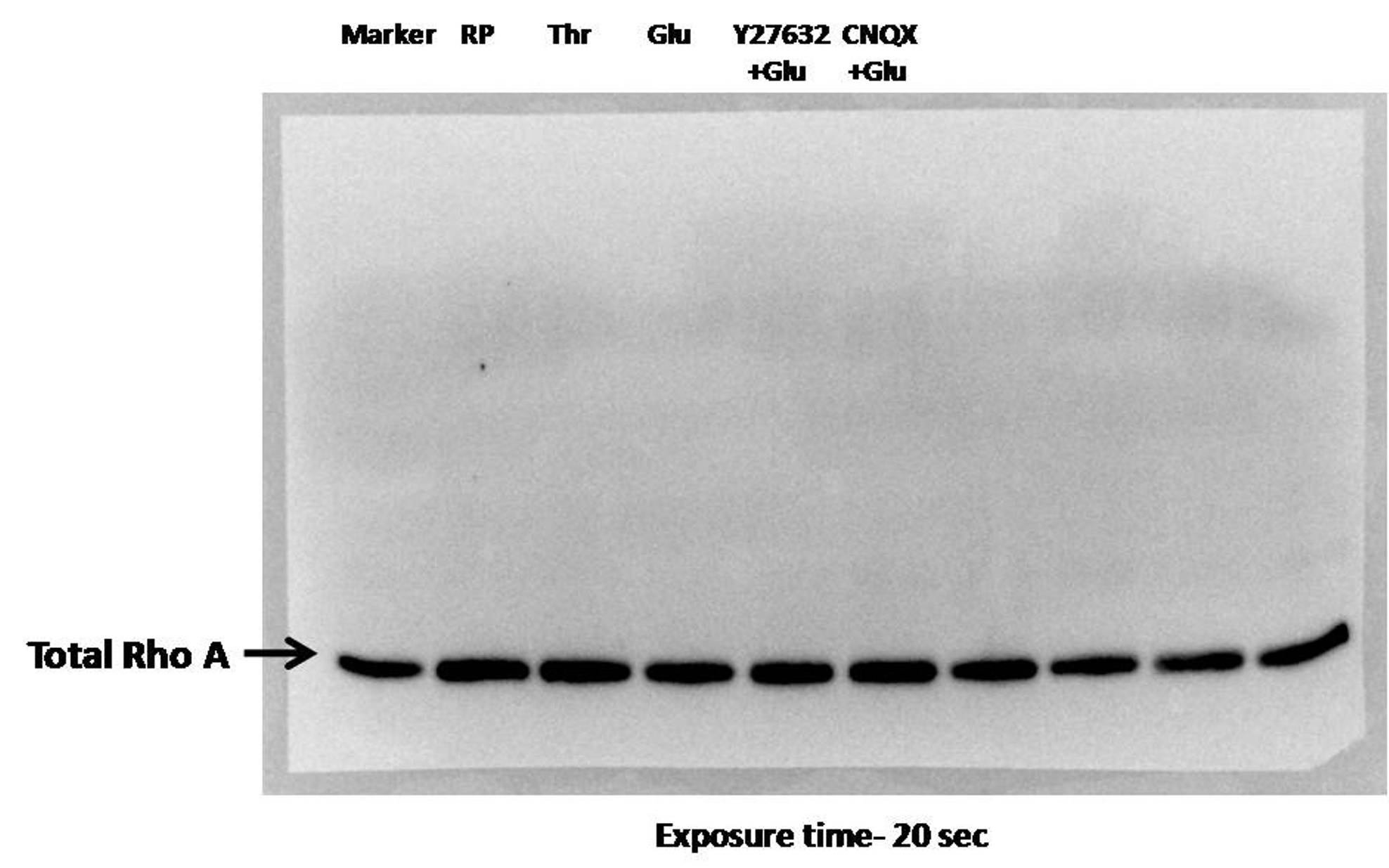


Supplementary Figure S4. Uncropped blots of figure 4D against RhoA-GTP (upper panel) and total RhoA (lower panel).


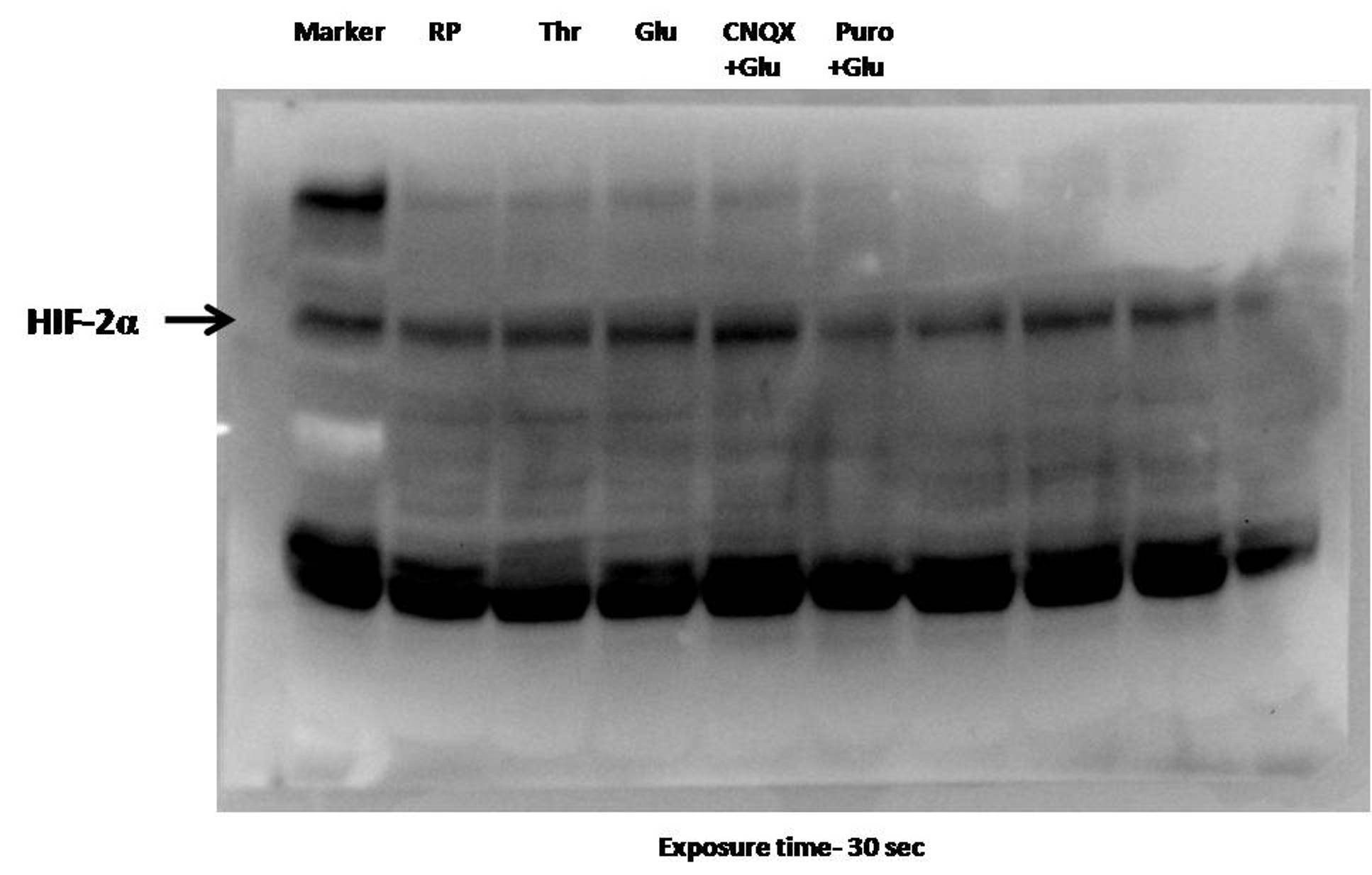


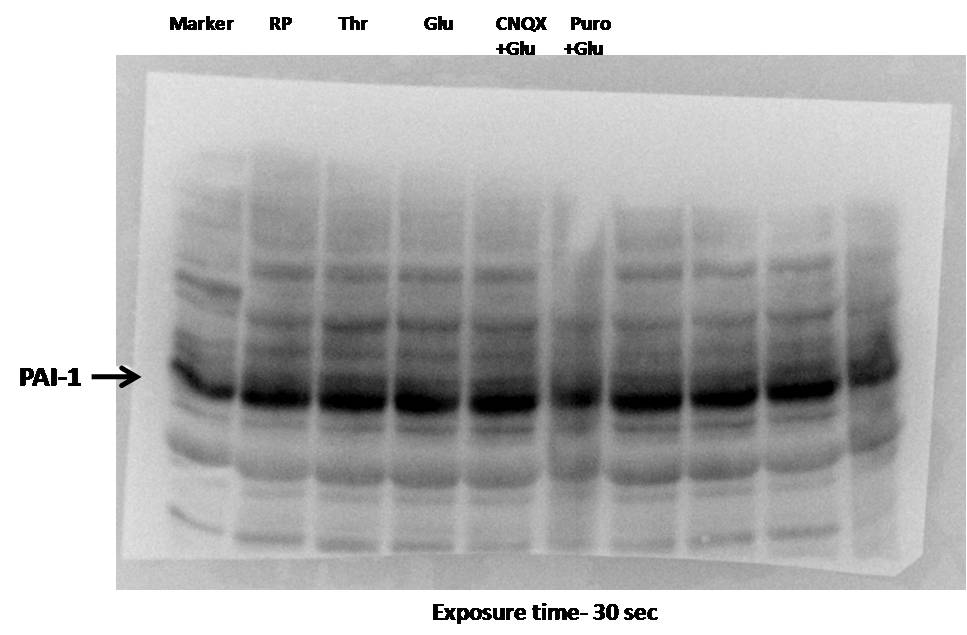


Supplementary Figure S5. Uncropped blots of figure 5A against protein HIF-2α (upper panel) and PAI-1 (lower panel).


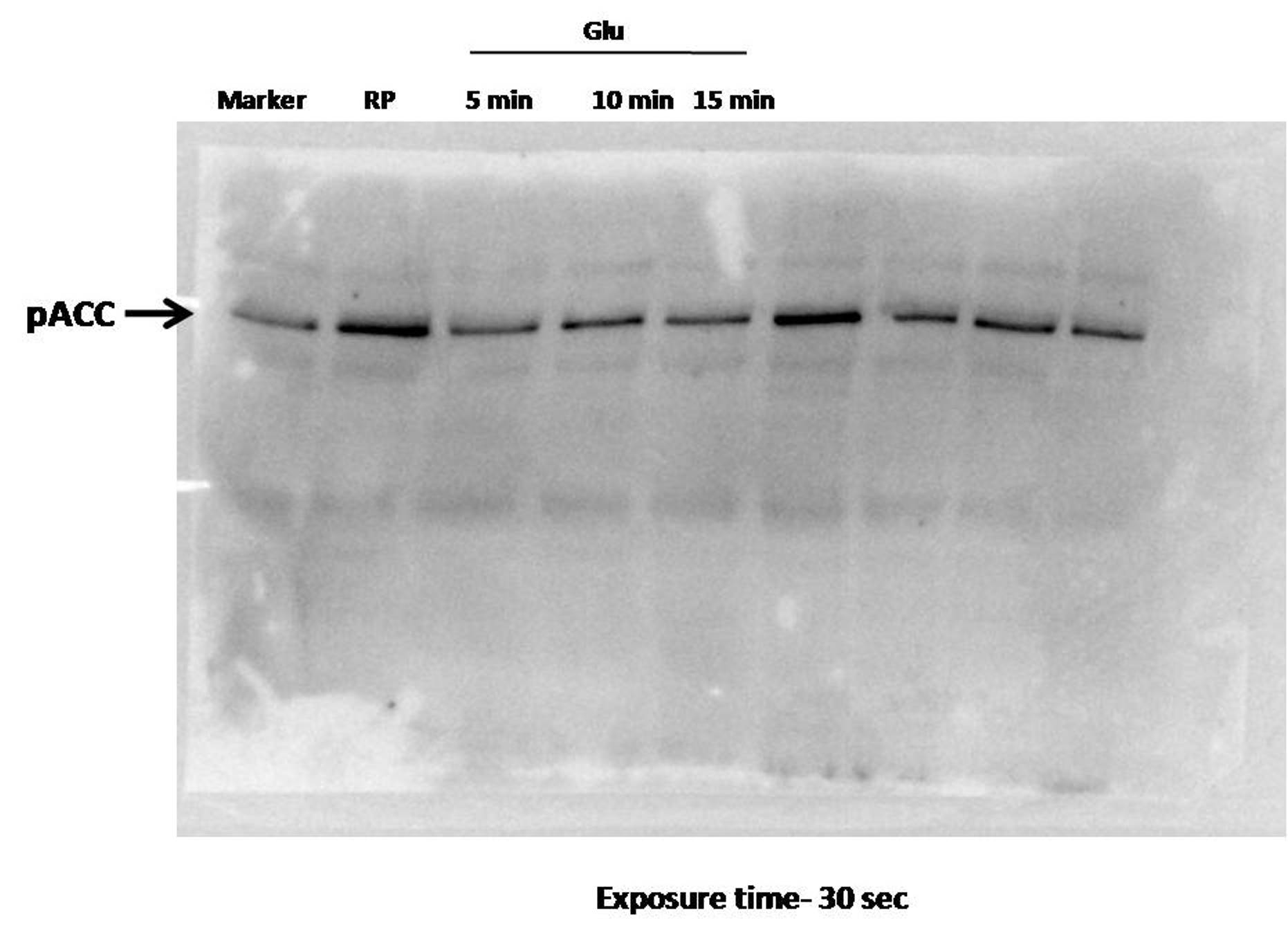

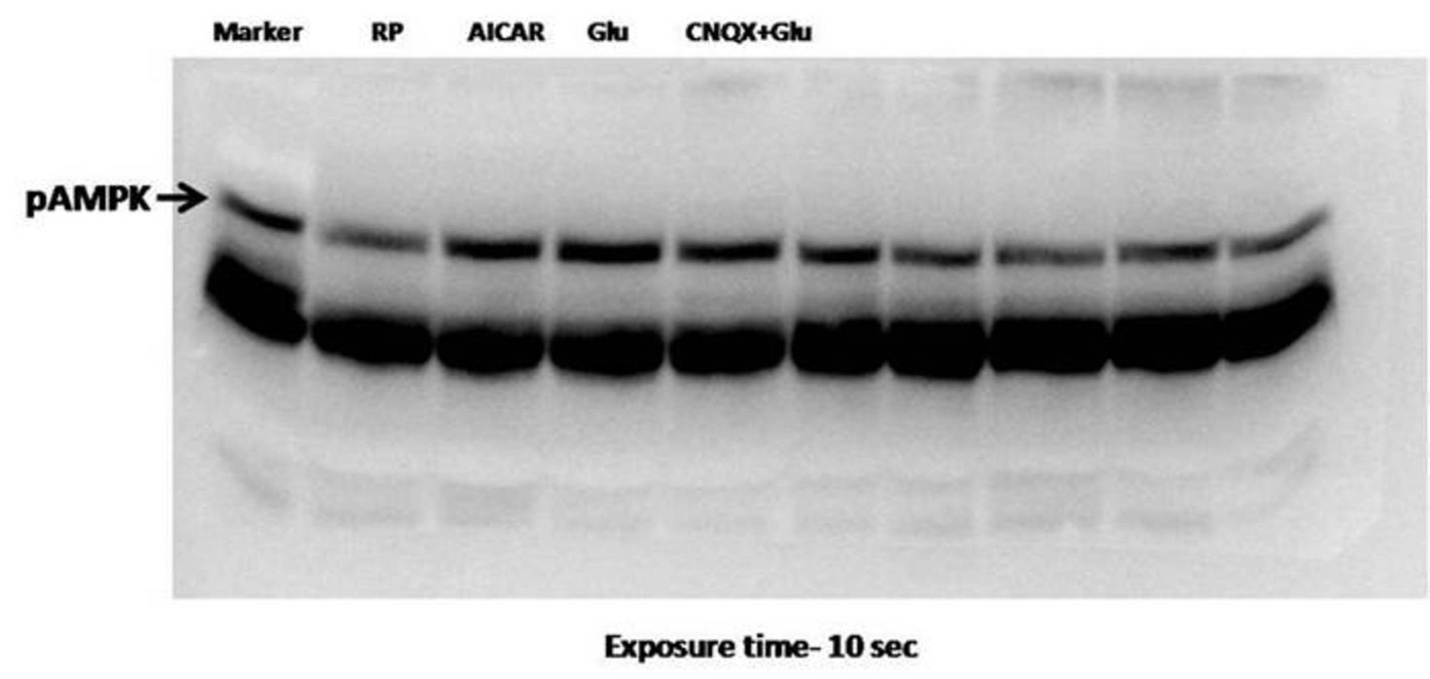


Supplementary Figure S6. Uncropped blots of figure 7A against protein pAMPK (upper panel) and pACC (lower panel).
